# The role of the corpus callosum in language network connectivity in children

**DOI:** 10.1111/desc.13031

**Published:** 2020-09-01

**Authors:** Lisa Bartha‐Doering, Kathrin Kollndorfer, Ernst Schwartz, Florian Ph.S. Fischmeister, Johanna Alexopoulos, Georg Langs, Daniela Prayer, Gregor Kasprian, Rainer Seidl

**Affiliations:** ^1^ Department of Pediatrics and Adolescent Medicine Medical University of Vienna Vienna Austria; ^2^ Comprehensive Center for Pediatrics Medical University of Vienna Vienna Austria; ^3^ Department of Biomedical Imaging and Image‐Guided Therapy Medical University of Vienna Vienna Austria; ^4^ Institute of Psychology University of Graz Graz Austria; ^5^ BioTechMed Graz Austria; ^6^ Department of Psychoanalysis and Psychotherapy Medical University of Vienna Vienna Austria

**Keywords:** corpus callosum, functional imaging, language development, language network

## Abstract

The specific role of the corpus callosum (CC) in language network organization remains unclear, two contrasting models have been proposed: inhibition of homotopic areas allowing for independent functioning of the hemispheres versus integration of information from both hemispheres. This study aimed to add to this discussion with the first investigation of language network connectivity in combination with CC volume measures. In 38 healthy children aged 6–12, we performed task‐based functional magnetic resonance imaging to measure language network connectivity, used structural magnetic resonance imaging to quantify CC subsection volumes, and administered various language tests to examine language abilities. We found an increase in left intrahemispheric and bilateral language network connectivity and a decrease in right intrahemispheric connectivity associated with larger volumes of the posterior, mid‐posterior, and central subsections of the CC. Consistent with that, larger volumes of the posterior parts of the CC were significantly associated with better verbal fluency and vocabulary, the anterior CC volume was positively correlated with verbal span. Thus, children with larger volumes of CC subsections showed increased interhemispheric language network connectivity and were better in different language domains. This study presents the first evidence that the CC is directly linked to language network connectivity and underlines the excitatory role of the CC in the integration of information from both hemispheres.

## Graphical abstract

In 38 healthy children aged 6–12, the present study measured language network connectivity, corpus callosum subsection volumes, and language abilities. Children with larger volumes of CC subsections showed increased interhemispheric language network connectivity and were better in different language domains. The present study suggests that the corpus callosum is directly linked to language network connectivity and underlines the excitatory role of the corpus callosum in the integration of information from both hemispheres. .

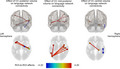

**Research Highlights**

- This study represents the first investigation of language network connectivity in combination with corpus callosum measures.
- Children with larger corpus callosum volumes have increased left intrahemispheric and interhemispheric language network connectivity.
- Children with larger corpus callosum volumes are better in different language domains.
- The corpus callosum plays an excitatory role in the integration of information of both hemispheres.

## 1 INTRODUCTION

The corpus callosum (CC) represents the major interhemispheric white matter tract and plays an important role in the transmission of signals between hemispheres (Paul et al., 2007). The CC is thought to influence language abilities through its involvement in the development of the functional network organization (Friederici & Alter, 2004), however, the specific role of the CC in the functional organization between hemispheres remains unclear. Although there is evidence for the association between CC structure and language abilities, no studies are available that directly link CC properties and the neural language network.

The association of CC structure and language abilities is well known from previous studies, where links between microstructural CC integrity and language abilities have been found (e.g. Chiang et al., 2009; Dunst, Benedek, Koschutnig, Jauk, & Neubauer, 2014), and size and volume of the CC have been related to language abilities (e.g. Hutchinson et al., 2009; Luders et al., 2011) in both adults and children. The direction of this association, however, is not consistent between studies: Whereas adult sample studies mostly reported positive associations between CC measures and verbal abilities (Chiang et al., 2009; Dunst et al., 2014; Luders et al., 2011; Strauss, Wada, & Hunter, 1994; but see Hutchinson et al., 2009), there is no clear consensus about the direction of this association in children and adolescents. Some pediatric studies showed decreased CC size and microstructural integrity in children to be associated with a higher verbal IQ, better reading performance, and better phonological awareness (Dougherty et al., 2007; Hutchinson et al., 2009; Luders et al., 2011). In contrast, other studies found increased structural and functional integrity of axonal CC fibers associated with verbal abilities (Aydin, Uysal, Yakut, Emiroglu, & Yilmaz, 2012; Deoni et al., 2016). In line with the latter, reduced CC volume and size have been reported in speech sound disorder (Luders et al., 2017), attention disorders (Langevin, Macmaster, Crawford, Lebel, & Dewey, 2014), and autism (Keary et al., 2009).

Studies that directly examine the association of callosal measures and language network connectivity are not available to date, thus, the specific role of the CC in the functional organization between hemispheres remains unclear. In general, two contrasting models have been suggested: According to the inhibitory model, fibers in the CC inhibit homotopic areas, allowing for independent functioning of the hemispheres (Cook, 1984). In contrast, the excitatory model proposes that the CC integrates information from both hemispheres, allowing improved interhemispheric connectivity (Galaburda, Rosen, & Sherman, 1990; Gazzaniga, 2000). In previous studies, connectivity has been indirectly inferred from language lateralization measures in several adult studies. In healthy adults, some studies have shown that a strong left lateralization of language is associated with higher anisotropic diffusion through the CC and increased callosal size, implying that in these individuals the two hemispheres are stronger interconnected, and thus supporting the inhibitory model (Hellige, Taylor, Lesmes, & Peterson, 1998; Josse, Seghier, Kherif, & Price, 2008; Karbe, Herholz, Halber, & Heiss, 1998; Westerhausen et al., 2006). However, other studies found less language lateralization associated with a greater size of the CC, consistent with the theory that a more bilateral network depends on increased interhemispheric connectivity through the CC, and thus favoring the excitatory model (Gootjes et al., 2006; Häberling, Badzakova‐Trajkov, & Corballis, 2011; Hines, Chiu, McAdams, Bentler, & Lipcamon, 1992; Westerhausen et al., 2004; Witelson, 1986; Yazgan, Wexler, Kinsbourne, Peterson, & Leckman, 1995).

Functional magnetic resonance imaging (fMRI) studies may add important information to the role of the CC in the development of language network connectivity. In general, fMRI connectivity studies are based on the assumption that positive connectivity reflects coordination and integration between hemispheres, whereas negative connectivity implies segregated or competing brain areas (Chu, Meltzer, & Bitan, 2018). However, to the best of our knowledge, no fMRI connectivity study is available that tests a possible association of language network connectivity and CC properties. Investigating healthy children provides a unique opportunity to inform our understanding of the role of the CC in the development of the language network. This study therefore investigated normalized CC volume, language‐task‐based functional connectivity, and language abilities in healthy school‐aged children with the aim to study the relationship between the volume of the CC and language network connectivity.

## 2 METHODS

### 2.1 Participants

Thirty‐eight healthy children (14 girls, 24 boys; *M*_age_ = 8.92 years, *SD* = 1.67) were recruited from community through flyers. All participants met the following criteria (a) no history of neurological disease and no clinical evidence of neurological dysfunction or developmental delay; (b) native, monolingual German speakers; (c) normal or corrected‐to‐normal vision and normal hearing; (d) age 6–12 years. We chose the age period of school‐age as it has shown to mark a distinctive period between major developmental transition points (National Research Council, 1984). All participants were attending regular school classes, and no study participant was on medication. Handedness ranged from +60 to +100 (*M* = 96.05, *SD* = 9.17), as measured with the Edinburgh Handedness Inventory EHI (Oldfield, 1971). Participants received a 30€ voucher for a book store. The studies were approved by the Ethics Committee of the Medical University of Vienna in accordance with the Helsinki Declaration of 1975. For children, age appropriate assent forms were provided, parents received a parental permission form. All children and one parent per child gave written informed consent prior to inclusion.

All children were investigated with structural MRI, fMRI, and neurolinguistic assessment.

### 2.2 MRI data acquisition

#### 2.2.1 FMRI language task

During fMRI assessment, the German version of an auditory description definition task was administered (Bartha‐Doering, Kollndorfer, et al., 2018; Bartha‐Doering, Novak, et al., 2018; Berl et al., 2014; Sepeta et al., 2016). In the auditory description definition condition, the participants heard the definition of an object followed by a noun and were instructed to press a button each time the definition truly described the noun. The control condition consisted of reverse speech, with some items additionally containing a pure tone at the end. The participants were instructed to press the button each time he/she heard the tone. The control condition was designed to control for first‐ and second‐order auditory processing, attention, and motor response (You et al., 2011). We used a block design composed of five language condition blocks alternating with five control task blocks. Each block lasted for 40 s and consisted of 10 sentences presented every 4 s. Total fMRI scan time was 6 min 40 s. Task performance was evaluated by the overall accuracy in the language condition and the control task separately. Detailed description of the fMRI paradigm can be found in Bartha‐Doering, Kollndorfer, et al. (2018), Bartha‐Doering, Novak, et al. (2018), or Bartha‐Doering et al. (2019).

Prior to MRI measurements, children were prepared for the MRI session with a video clip and a training session. The video showed the MRI setting and presented the MRI noise and followed a child from entrance to the MRI institute until the scanning procedure. The training session comprised 10 items for each task.

#### 2.2.2 MRI image acquisition

All participants were scanned on a 3T Siemens TIM Trio (Siemens Medical Solutions) and equipped with a high‐performance gradient system to support fast, high‐resolution whole‐brain echo‐planar imaging. 3D structural MRI scans were performed using an isocubic magnetization‐prepared rapid gradient‐echo (MPRAGE, T1‐weighted, TE/TR _ 4.21/2,300 ms, inversion time 900, with a matrix size of 240 × 256 × 160, voxel size 1 × 1 × 1.10 mm, flip angle 9°) sequence. FMRI was acquired using a phase corrected blipped gradient echo, single shot echo planar imaging (EPI) sequence. Altogether, 200 EPI volumes were acquired with a square field of view of 210 mm, voxel size 2.1 × 2.1 × 4 mm, 25% gap and 20 slices aligned parallel to the AC‐PC plane using a repetition time (TR) of 2,000 ms, echo time (TE) 42 ms, and a flip angle of 90.

### 2.3 Language examinations

Verbal abilities were assessed using standardized tests of vocabulary, verbal memory, and verbal fluency. Test was chosen which examine functions important for language consolidation and vocabulary growth (Deák, 2014), and which are sensitive enough to depict subtle variations in normal cognitive functioning (Thornton & Lukas, 2012). Expressive vocabulary was examined using the Wortschatz‐ und Wortfindungstest WWT (Glück, 2011). This test provides information about expressive vocabulary in different lexical categories including nouns, verbs, and adverbs/adjectives. Immediate verbal auditory attention, short‐term, and working memory were investigated by the digit span forward and backwards tasks of the Hamburg‐Wechsler‐Intelligenztest für Kinder IV (Petermann & Petermann, 2011). Verbal learning was assessed with the German version of the Auditory Verbal Learning Test (Lezak, 1995), the Verbaler Lern‐ und Merkfähigkeitstest (Helmstaedter, Lendt, & Lux, 2001). This test measures the learning efficiency of a list of words, short‐term recall after distraction, long‐term recall, and recognition. Verbal fluency was evaluated using the Regensburger Wortflüssigkeitstest (Aschenbrenner, Tucha, & Lange, 2001) which requires the participant to name, within 2 min, as many words as possible of the semantic category animals. Overall, seven test scores of different language functions were obtained.

### 2.4 Data analysis

#### 2.4.1 CC volume calculation

**FIGURE 1:**
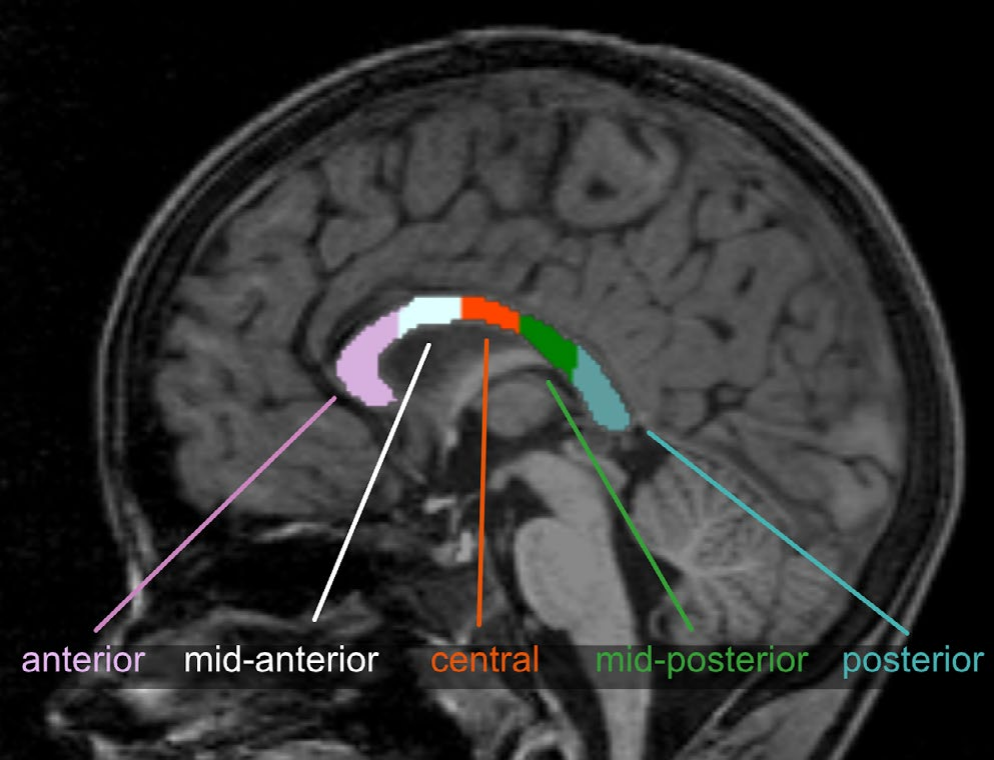
Segmentation of the corpus callosum into five subsections with FreeSurfer in a healthy study participant. Figure design inspired by Goldman et al. (2017)

MRI scans were processed with FreeSurfer version 6.0 software (http://freesurfer.net). The CC was identified and separated along its primary eigenaxis into anterior, mid‐anterior, central, mid‐posterior, and posterior segments with Freesurfer's automatic labeling (Fischl et al., 2002; Rosas et al., 2010; Figure 1). Subsection volumes and total intracranial volumes were computed in Freesurfer. Subsection volumes were normalized by dividing each volume by the individual total intracranial volume to control for individual variations in brain size.

#### 2.4.2 Task‐based connectivity analysis

Functional connectivity was analyzed using the CONN toolbox 18b (Whitfield‐Gabrieli & Nieto‐Castanon, 2012) running on SPM12 (Wellcome Department of Cognitive Neurology) using MATLAB (Version 9.1; Mathworks, Inc.). Preprocessing of functional data was done using recommended default settings (functional realignment and unwarping, slice‐time correction, ART‐based outlier detection, direct segmentation and MNI normalization, and smoothing with an 8 mm Gaussian kernel). Temporal fluctuations of segmented white matter and cerebrospinal fluid were identified within CONN using the aCompCor method (Behzadi, Restom, Liau, & Liu, 2007) and finally regressed together with realignment parameters and main task effects from the preprocessed single voxel data. Finally, cleaned data were band‐pass filtered between 0.008 and 0.09 Hz. For the following seed‐based ROI‐to‐ROI analysis bivariate correlation was used to determine the temporal associations between each of the ROI‐to‐ROI functional connections and then Fisher's *z* transformed. Second‐level regression analyses were performed to calculate the effect of the normalized CC subsection volumes on the network connectivity during language processing. Significance level was set at *p*_FDR_ < .05 (False Discovery Rate corrected).

#### 2.4.3 ROI selection

**FIGURE 2:**
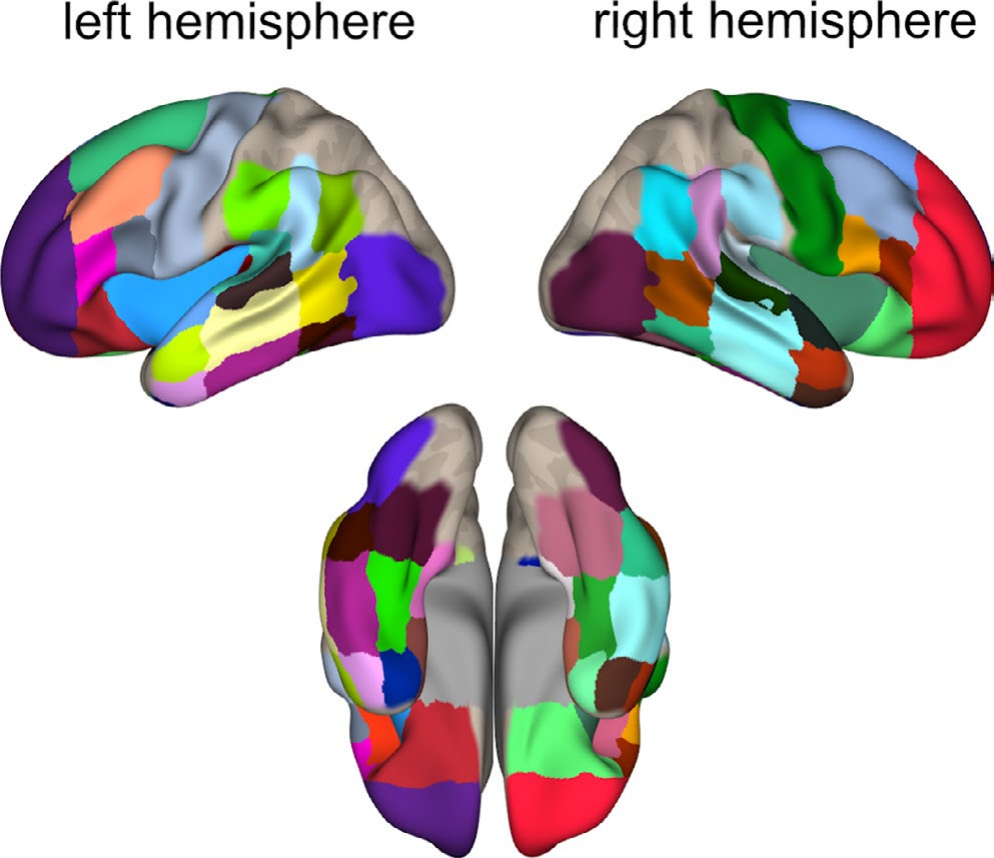
Region of interests selected for this study

To define the language network for task‐based connectivity analysis, all ROIs were selected from the Brainnetome Atlas (Fan et al., 2016) that were characterized as involved in language processing, along with their contralateral homologues. The Brainnetome Atlas uses meta data labels of the BrainMap Database (www.brainmap.org/taxonomy) using forward and reverse inferences (Cieslik et al., 2013; Clos, Amunts, Laird, Fox, & Eickhoff, 2013; Eickhoff et al., 2011). For our language nodes, we included regions that were involved in paradigms of speech, semantics, syntax, and phonology, while we excluded regions that were only involved in orthography. In addition, we included the hippocampi and parahippocampal gyri within both hemispheres as their involvement in semantic language processing was shown in previous research (Bartha et al., 2003, 2005; Bartha‐Doering, Novak, et al., 2018). In sum, we obtained 29 ROIs within each hemisphere and a total of 58 ROIs (Figure 2).

#### 2.4.4 Language tests analysis

Raw scores of language tests were transformed into age‐adjusted *z*‐scores for each test. For the WWT vocabulary norms are only available until 10;11 years of age. We therefore transformed the WWT raw scores of the children aged 11–12 (*n* = 5) into *z*‐scores based on the 10;11‐year‐old children with the risk of an overestimation of WWT results in these participants. An overall verbal *z*‐score was calculated from the mean of all seven test *z*‐scores. In line with clinical conventions, individual percentile ranks from *SD* −1 to *SD* 1 were defined within the average range. Overall verbal performance below *SD* −1 was read as below average, and performance below *SD* −2 was interpreted as reduced.

#### 2.4.5 Further statistical analysis

Further statistical analyses were conducted using SPSS Statistics (version 25). The strength of the relationship between language test scores and callosal subsection volumes with age was evaluated using Spearman's rank correlation. Mann–Whitney *U* test was used to examine if language test scores or callosal subsection volumes differed by sex. Significance of correlations was set based on a strict Bonferroni correction factor, that is, *α* =.05/number of comparisons, resulting in a *p*_corr_ <.006. The relationship between CC subsection volumes and language test *z* scores was evaluated using regression analyses. Separate regression models were performed for each language test. In each model, age was included as forced‐entry independent variable, after which callosal subsection volumes were entered as independent variables with a stepwise method.

## 3 RESULTS

### 3.1 CC subsection volumes results

Results of volumetric analyses are presented in Table 1. Normalized CC central subsection volume significantly correlated with age, whereas the other subsections did not show a significant relationship with age. There were no differences in CC subsection volumes by gender.

**TABLE 1:** Normalized CC subsection volumes, correlations with age, and differences by gender

	% intracranial volume *M* (*SD*)	Age *r*_s_ (*p*)	Gender *p*
CC posterior	0.054 (0.008)	.23 (.168)	.823
CC mid‐posterior	0.030 (0.005)	.25 (.133)	.800
CC central	0.034 (0.009)	.50 (.001)*	.501
CC mid‐anterior	0.031 (0.008)	.34 (.036)	.917
CC anterior	0.056 (0.007)	.10 (.544)	.643

Note

Uncorrected *p*‐values are given, statistical significance after Bonferroni correction (*p*_corr_ < .01) is indicated with *.

Abbreviation: CC, corpus callosum.

### 3.2 In‐scanner task performances

On‐site check of in‐scanner performance showed adequate response during the fMRI paradigm in all participants. Unfortunately, due to technical reasons, task accuracy for the in‐scanner performance was missing in 17 participants. Mean correct response in 21 study participants was 91.24% (*SD* = 9.11) for the auditory description definition condition and 91.71% (*SD* = 10.83) for the tone condition. Overall, these data indicate good task performances. However, due to the large amount of missing data, we waived further analyses of in‐scanner task performances.

### 3.3 Verbal test results

Language testing revealed average overall verbal abilities in 33 children, below average language abilities in four children, and above average abilities in one child (overall *M* = −0.03, *SD* = 0.61, range = −1.46 to 1.06). None of the children showed reduced overall language abilities (overall *z*‐score *SD* below −2). Single language test results were more heterogeneous, their results point to a wide distribution of language performances in the study group (Table 2). Spearman correlation analysis revealed a significant correlation between age and verbal recognition (*r*_s_ = .49, *p* = .002), though performance was already age‐corrected. Thus, compared to their specific age groups, older participants in our study were better in verbal recognition than younger ones. In addition, an association of age with verbal fluency and verbal span was observed, but significance did not survive correction for multiple comparisons. Verbal performance did not differ by gender.

**TABLE 2:** Language test results, correlations with age, and differences by gender

	Mean *z*‐score (*SD*)	Age *r*_s_ (*p*)	Gender *p*
Overall verbal abilities	−0.03 (0.61)	.37 (.022)	.463
Expressive vocabulary	0.42 (1.18)	.15 (.378)	.709
Verbal span	0.03 (0.96)	.33 (.045)	.445
Verbal learning efficiency	−0.44 (1.05)	.21 (.216)	.120
Verbal short‐term memory	0.16 (0.91)	.07 (.657)	.601
Verbal long‐term memory	0.30 (0.85)	.03 (.878)	.144
Verbal recognition	−0.45 (0.98)	.49 (.002)*	.964
Verbal fluency	−0.21 (1.15)	.41 (.013)	.963

Note

Uncorrected *p*‐values are given, statistical significance after Bonferroni correction (*p*_corr_ < .006) is indicated with *.

### 3.4 Relationship between CC volume and language network connectivity

**FIGURE 3:**
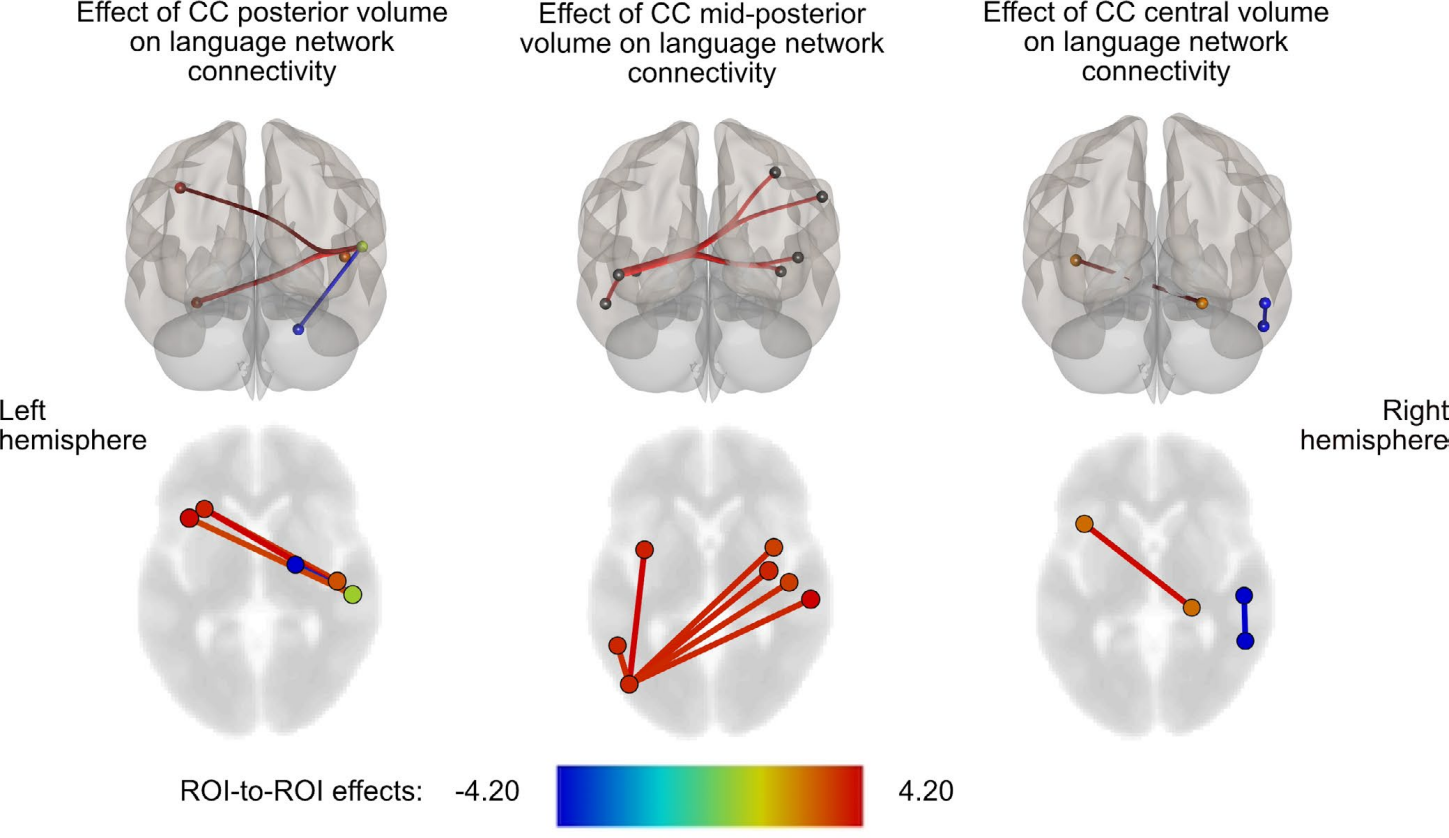
The effect of CC subsection volumes on language network connectivity. ROI‐to‐ROI analyses (*p*_FDR_ seed‐level correction < .05) revealed an increase in the bilateral language network connectivity (red colors) and a decrease in right intrahemispheric connectivity (blue colors) with larger volumes of the posterior, mid‐posterior, and central subsections of the CC. The volumes of the anterior CC subsections did not show a significant association with language network connectivity. Brain images are displayed in neurological convention. CC, corpus callosum; ROI, region of interest

ROI‐to‐ROI analyses revealed a significant effect of normalized CC subsection volumes on language network connectivity (Figure 3; Table 3). A larger posterior CC volume was significantly associated with increased connectivity between the left frontal orbital cortex and right planum temporale and Heschl's gyrus of the right hemisphere, as well as between the left middle frontal gyrus and the right planum temporale. Increased posterior CC volume was furthermore significantly related to a decreased connectivity within the right hemisphere between the planum temporale and the anterior part of the parahippocampal gyrus. A larger mid‐posterior CC volume significantly correlated with increased connectivity between the left inferior occipital cortex and the left insular cortex, and between the left inferior frontal cortex and the insular cortex, precentral gyrus, supramarginal gyrus, Heschl's gyrus, and inferior temporal gyrus of the right hemisphere. A larger central CC volume was significantly associated with increased connectivity between the left operculum and the right parahippocampal gyrus, as well as with decreased connectivity within the right hemisphere between the posterior and the temporo‐occipital parts of the inferior temporal gyrus. In contrast, mid‐anterior and anterior CC subsection volumes were not significantly associated with language network connectivity.

**TABLE 3:** Effect of CC subsection volumes on language network connectivity

ROI to ROI	*T*	*p*_FDR_
Effect of posterior CC volume on language network connectivity		
Connectivity increases		
Frontal orbital cortex L – planum temporale R	4.20	.009
Frontal orbital cortex L – Heschl's gyrus R	3.50	.035
Middle frontal gyrus L – planum temporale R	3.35	.035
Connectivity decreases		
Planum temporal R – parahippocampal gyrus, anterior R	−3.64	.023
Effect of mid‐posterior CC volume on language network connectivity		
Connectivity increases		
Inferior occipital cortex, lateral L – insular cortex L	3.74	.033
Inferior occipital cortex, lateral L – precentral gyrus R	3.35	.033
Inferior occipital cortex, lateral L – insular cortex R	3.30	.033
Inferior occipital cortex, lateral L – supramarginal gyrus, anterior R	3.26	.033
Inferior occipital cortex, lateral L – Heschl's gyrus R	3.24	.033
Inferior occipital cortex, lateral L – inferior temporal gyrus, temporo‐occipital L	3.20	.033
Connectivity decreases	n.s.	
Effect of central CC volume on language network connectivity		
Connectivity increases		
Frontal operculum L – paraphippocampal gyrus, posterior R	3.70	.039
Connectivity decreases		
Inferior temporal gyrus, posterior R – inferior temporal gyrus, temporo‐occipital R	−3.81	.029
Effect of mid‐anterior CC volume on language network connectivity	n.s.	
Effect of anterior CC volume on language network connectivity	n.s.	

Abbreviations: CC, corpus callosum; L, left; R, right; ROI, region of interest.

In sum, children with larger normalized volumes of the central and posterior CC exhibited increased left intrahemispheric and interhemispheric connectivity and decreased right intrahemispheric connectivity within language‐associated brain areas.

### 3.5 Relationship between CC volumes and language test results

Normalized CC subsection volumes were not significantly different by gender, but the central subsection volume significantly correlated with age (*r*_s_ = .50, *p* = .001). Other subsection volumes were not significantly associated with age (posterior CC volume *r*_s_ = .23, *p* = .168; mid‐posterior CC volume *r*_s_ = .25, *p* = .133; mid‐anterior CC volume *r*_s_ = .34, *p* = .036; anterior CC volume *r*_s_ = .10, *p* = .544).

Using a hierarchical linear regression model with age forced entered on the first step as covariate, we found significant associations among normalized CC subsection volumes and different language domains *z*‐scores. Specifically, the posterior subsection volume of the CC was significantly associated with verbal fluency (*F*_2,35_ = 6.608 *p* = .004, adjusted *R* ^2^ = 0.280; posterior subsection volume *β* = .389, *p* = .013) and with vocabulary (*F*_2,35_ = 3.466, *p* = .042, adjusted *R* ^2^ = 0.118; posterior subsection volume *β* = .372, *p* = .025). The anterior CC volume was associated with verbal span (*F*_2,35_ = 7.202, *p* = .002, adjusted *R* ^2^ = 0.292; anterior subsection volume *β* = .358, *p* = .018). No significant associations between the memory domains and CC subsection volumes were found.

## 4 DISCUSSION

This study presents the first evidence that the CC is directly linked to language network connectivity. We investigated task‐based fMRI, CC subsection volumes, and language abilities in healthy, school‐aged children. We found an increase in left intrahemispheric and bilateral language network connectivity and a decrease in right intrahemispheric connectivity associated with larger volumes of the posterior, mid‐posterior, and central subsections of the CC. Consistent with that, larger volumes of the posterior parts of the CC were significantly associated with better verbal fluency and vocabulary, the anterior CC volume was positively correlated with verbal span. In sum, children with larger volumes of CC subsections showed increased interhemispheric language network connectivity and were better in different language domains.

### 4.1 Interhemispheric language network connectivity associated with CC volume

Our study supports the excitatory model proposing CC as the pathway that integrates information from both cerebral hemispheres, allowing improved interhemispheric connectivity and better language functioning (Galaburda et al., 1990; Gazzaniga, 2000). Integration between the two hemispheres may increase the amount of cortex volume that can be devoted to a particular cognitive task (Bloom & Hynd, 2005; Yazgan et al., 1995). Studies have shown that increased task difficulty results in increased activation volume in bilateral perisylvian regions (Caplan et al., 2002; Just, Carpenter, Keller, Eddy, & Thulborn, 1996; Just & Varma, 2007; Kaan & Swaab, 2002), thus, interaction between hemispheres may be especially beneficial under conditions of high complexity and attentional demand (Banich & Brown, 2000). In fact, language is one of the highest cognitive domains, and the fMRI paradigm that we used in this study is a demanding language task that particularly involves comprehension of a phrase, recall, and decision, in addition to attentional and auditory working memory abilities (Bartha‐Doering, Kollndorfer, et al., 2018). This study shows that the efficiency of this complex language network depends upon the callosal pathways integrating information from both hemispheres.

Within a bilateral language network, one might expect the right homologues of typical language‐associated regions to be strongly engaged. Interestingly, increased interhemispheric network connectivity associated with larger CC volumes almost exclusively involved mesial and lateral *temporal* regions in the right hemisphere, whereas right inferior frontal or parieto‐occipital regions were not stronger integrated in the bilateral language network. However, keeping in mind the specific language decision task in our study, right hippocampal and lateral temporal regions might support highly efficient word retrieval. Right temporal activations during semantic priming and semantic decision have been reported in healthy children and adults (Bartha et al., 2003, 2005; Geukes et al., 2013), and the importance of right mesial and lateral temporal regions for word retrieval has been underlined in several studies in neurological patients (Bonelli et al., 2012; Schwartz et al., 2009; Trebuchon‐Da Fonseca et al., 2009).

Besides an increase in left hemisphere connectivity as well as interhemispheric connectivity, we found a decrease in connectivity between mesial and lateral temporal areas in the right hemisphere associated with larger CC volumes. A possible explanation for this finding might be that language abilities profit from additional right temporal language processing that support and strongly interact with left hemisphere processing. However, these right‐sided language regions seem to play a subordinate role, and a more independent and stronger intrahemispheric interplay between them might not be beneficial for language efficiency in right‐handed individuals. This hypothesis is in line with studies on aphasia recovery that shows an increase in right hemisphere recruitment in early recovery, but a reduction over time in association with clinical improvements (Breier et al., 2009; Fernandez et al., 2004; Kurland et al., 2008; Saur et al., 2006).

### 4.2 Verbal abilities associated with CC volume

In line with the excitatory model, CC subsection volumes were not only associated with language network connectivity, but also positively related to language abilities within this study. A positive correlation between verbal abilities and CC size or volume has previously been reported in children and adolescents with neurological diseases: Preterm born children exhibit smaller CC subsections associated with lower verbal intelligence measures (Caldu et al., 2006; Narberhaus et al., 2007; Nosarti et al., 2004; Peterson et al., 2000). Significantly reduced dimensions of the CC have been observed in children with speech sound disorder, developmental language disorders, and autism (Keary et al., 2009; Langevin et al., 2014; Luders et al., 2017).

On the contrary, Dougherty et al. (2007) found negative correlations of phonological awareness with diffusivity and fractional anisotropy of callosal fibers. They suggested that children with better phonological skills have reduced interhemispheric connectivity. However, several physiological mechanisms could cause this difference, and the results of this study may also be interpreted in a different way, as the authors state themselves. Good performers may have a higher proportion of large axons and thus a lower density of cell membranes passing through the callosum. A different explanation is that the membranes and myelin sheaths are more permeable to diffusing water in children with better phonological skills (Dougherty et al., 2007). Overall, DTI measurement provide an excellent way to demonstrate fiber tracts in the white matter, but various methodological aspects, spatial resolution, and differential effects of various tissue properties require a cautious interpretation (Zilles & Amunts, 2015).

In additon, Hutchinson et al. (2009) found a higher performance IQ associated with a smaller size of the posterior CC regions in adolescents and young adults 14–25 years of age. Similarily, Luders et al. (2011) reported negative correlations between callosal thickness in the splenium and intellectual abilities in a group of healthy children and adolescents aged 7–17 years. Yet, the same study found that within the large age range of their study population, younger children exhibited mainly positive correlations between callosal thickness and intellectual abilities. Accordingly, Deoni et al. (2016) investigated a larger sample of typically developing children and reported that the profile of white matter myelination across the first 5 years of life was specifically related to cognitive abilities. These findings together with ours in children between 6 and 12 years of age point to age‐related differences in the relationship of CC properties and cognition. Such age‐related differences have been attributed to the maturational gradient of the CC and age‐related changes in task demands (Hutchinson et al., 2009).

It may be hypothesized that earlier and/or more intense language experience leading to elaborated language abilities may affect the callosal fibers interconnecting developing language regions. In fact, some studies showed that (early) cognitive experience may affect the myelination of the CC: Musicians who had begun their musical training before the age of seven had a larger anterior CC compared to musicians beginning later (Schlaug, Jancke, Huang, Staiger, & Steinmetz, 1995), and bilinguals exhibited a significantly larger mid‐anterior segment of the CC compared to monolinguals (Felton et al., 2017).

CC volume has shown to depend on the number of callosal fibers, with more axons and increased axonal diameters being associated with larger CC volumes (Luders et al., 2017; Riise & Pakkenberg, 2011). Information transfer of premotor and prefrontal areas between both hemispheres involves the anterior CC, and transfer between temporal, parietal, and occipital regions routes through the posterior CC (Fabri, Pierpaoli, Barbaresi, & Polonara, 2014; Zarei et al., 2006). This is very well in accordance with this study, where the anterior CC volume was significantly associated with verbal memory span, a cognitive function known to largely involve bilateral frontal brain areas (Emch, von Bastian, & Koch, 2019). Furthermore, the posterior subsection volumes of the CC were significantly associated with verbal (semantic) fluency and vocabulary. These semantic language abilities also rely on frontal areas, but primarily involve mesial and lateral temporal areas of both hemispheres (Bartha‐Doering, Kollndorfer, et al., 2018; Bartha‐Doering, Novak, et al., 2018).

### 4.3 Functional connectivity reflects structural connectivity?

One of the well – established beliefs of CC connectivity is its structural connection of homologues. The rostrum and genu connect frontal homologues interhemispherically, the splenium connects occipital, parietal, and temporal homologues, and the body and isthmus are thought to connect (pre)motor and primary sensory regions between hemispheres. It is often assumed that functional connectivity reflects structural brain connectivity. The results presented here thus seem to violate the well‐established principle of homotopic CC connectivity, as we found a larger posterior CC volume associated with increased functional connectivity between left frontal and right temporal areas. However, in addition to the homotopic structural connections through the CC, there is a sizeable fraction of heterotopic transcallosal projections (Chovsepian, Empl, Correa, & Bareyre, 2017). These are a subset of the contralateral counterpart of ipsilateral associative connections (Hedreen & Yin, 1981; Mancuso, Uddin, Nani, Costa, & Cauda, 2019). However, above all, the relationship between structure and function is not straightforward (Damoiseaux & Greicius, 2009; Mancuso, Costa, et al., 2019; Mancuso, Uddin, et al., 2019). Functional connectivity strength correlates with structural connectivity strength, but functional networks often exceed patterns of structural connectivity (Adachi et al., 2012). Functional connectivity can sometimes even be observed between regions without structural connectivity, indicating functional correlations that are mediated by structural connections via a third region (Damoiseaux & Greicius, 2009). Contrary to structural connectivity, functional connectivity can furthermore be positive or negative and can thus provide additional information of the nature of connectivity between areas (Fox et al., 2005).

### 4.4 The developmental aspect of these findings

We chose the age period between 6 and 12 years as it marks a distinctive period between major developmental transition points (National Research Council, 1984). Within the developmental period of 6–12 years, the children acquire written language and start using abstract language (Vigliocco, Ponari, & Norbury, 2018). After these 12 years, basic language acquisition is completed, the rate of vocabulary acquisition slows down (Rice & Hoffman, 2015), and white matter growth has shown to decelerate (Arain et al., 2013; Thompson et al., 2000).

We found a significant correlation of age with some volumetric measures in this study and addressed this point by controlling the analyses for age. However, age may nevertheless be a factor influencing the relationship between CC properties, language network, and language abilities, and this relationship may change during adolescence. Hence, the results reported here are only valid for the respective age group, more longitudinal research is necessary to investigate the trajectories of language network development during adolescence.

We aimed at investigating healthy children during the school‐age period, with ‘healthy’ defined as the absence of a neurological disease and no clinical evidence of neurological dysfunction or developmental delay. In line with clinical conventions (World Health Organization, 2010), their overall language performance was interpreted as reduced when the overall test score fell below two standard deviations of the child's age. None of the children presented overall language abilities below this threshold. Four children, however, performed below one standard deviation of the mean. Their overall language performance may thus be interpreted as below average. Some researchers, however, interpret scores below one standard deviation as delayed (Shulman & Capone, 2010). Thus, though these children were defined as healthy with no clinical evidence of developmental delay, their detailed standardized testing revealed below average, or dependent on the interpretation, even delayed language development. This has to be taken into account when interpreting the results of the study.

## 5 LIMITATIONS

Recording of in‐scanner task accuracy was missing due to technical reasons in many participants. While on‐site check of in‐scanner performance showed adequate response during the fMRI paradigm in all participants, the investigation of the relationship between in‐scanner performance, out‐scanner performance, and language network may have provided important additional information. Furthermore, there may have been an individual bias to respond or not respond during the fMRI task. Although performances in the recorded children were very high (mean above 90% correct), a response bias cannot be excluded.

## 6 CONCLUSIONS

This study for the first time provides evidence for a direct link between CC volume and language network connectivity in children. It underlines the excitatory role of the CC in fostering the integration of language information from both hemispheres. During development, the integration of right mesial and lateral temporal areas in the language network seems to be beneficial for language abilities; however, right‐sided language regions play a subordinate role within the language network.

## CONFLICT OF INTEREST

We have no conflict of interest to declare.

## Data Availability

Data available on request due to privacy and ethical restrictions.
